# Synergisms of genome and metabolism stabilizing antitumor therapy (GMSAT) in human breast and colon cancer cell lines: a novel approach to screen for synergism

**DOI:** 10.1186/s12885-020-07062-2

**Published:** 2020-07-02

**Authors:** Jérôme Ruhnau, Jonas Parczyk, Kerstin Danker, Britta Eickholt, Andreas Klein

**Affiliations:** Charité – Universitätsmedizin Berlin, Corporate Member of Freie Universität Berlin, Humboldt-Universität zu Berlin, and Berlin Institute of Health, Institute of Biochemistry, Charitéplatz 1, 10117 Berlin, Germany

**Keywords:** Synergism, drug combination, cancer therapy, Nutlin-3, PX-478, Dichloroacetate, NHI-2, MDA-MB-231, MCF-7, HT-29

## Abstract

**Background:**

Despite an improvement of prognosis in breast and colon cancer, the outcome of the metastatic disease is still severe. Microevolution of cancer cells often leads to drug resistance and tumor-recurrence. To target the driving forces of the tumor microevolution, we focused on synergistic drug combinations of selected compounds. The aim is to prevent the tumor from evolving in order to stabilize disease remission. To identify synergisms in a high number of compounds, we propose here a three-step concept that is cost efficient, independent of high-throughput machines and reliable in its predictions.

**Methods:**

We created dose response curves using MTT- and SRB-assays with 14 different compounds in MCF-7, HT-29 and MDA-MB-231 cells. In order to efficiently screen for synergies, we developed a screening tool in which 14 drugs were combined (91 combinations) in MCF-7 and HT-29 using EC_25_ or less. The most promising combinations were verified by the method of Chou and Talalay.

**Results:**

All 14 compounds exhibit antitumor effects on each of the three cell lines. The screening tool resulted in 19 potential synergisms detected in HT-29 (20.9%) and 27 in MCF-7 (29.7%). Seven of the top combinations were further verified over the whole dose response curve, and for five combinations a significant synergy could be confirmed. The combination Nutlin-3 (inhibition of MDM2) and PX-478 (inhibition of HIF-1α) could be confirmed for all three cell lines. The same accounts for the combination of Dichloroacetate (PDH activation) and NHI-2 (LDH-A inhibition). Our screening method proved to be an efficient tool that is reliable in its projections.

**Conclusions:**

The presented three-step concept proved to be cost- and time-efficient with respect to the resulting data. The newly found combinations show promising results in MCF-7, HT-29 and MDA-MB231 cancer cells.

## Background

### Introduction

Although a lot of progress has been made in the research of potential anti-cancer agents over the last decade, secondary therapy failure and disease progression is still the major problem in most tumor entities especially in the metastatic state of solid tumors [1, 2]. The tumor microevolution gives constantly rise to new populations of cancer cells with diverse properties [3] making it difficult to target them. Therefore, we developed a combinatory therapeutic approach that targets the tumor microevolution and its driving forces.

Industrial funds become more important in research. As industrial funding [4] and the focus on commercial interests increase, research is favourably conducted on newly bioengineered and patentable drugs [5] rather than generic compounds. Therefore, we aimed to establish a cost-efficient screening strategy that is feasible for independent work groups. In order to screen a relatively high number of potential compounds for their synergistic potency, we present here a three-step approach including a minimalistic drug interaction screening (MDIS) that is cost-efficient and can easily be established with basic laboratory equipment independent of expensive high-throughput devices.

### The tumor microevolution and its driving forces

Unfortunately, initial antitumor treatment frequently leaves residual disease from which the tumor regrows [6]. Microevolution of cancer cells often leads to drug resistance and tumor recurrence [7]. Important driving forces of the microevolution are the genomic instability [8], the tumor metabolism [9, 10] and a deregulated cell cycle [11] that converge in a high proliferation rate combined with a high occurrence of mutations. To treat such complex diseases, combinations of drugs that target different aspects of the disease and at best, act synergistically may be the method of choice. Another complex disease that can currently be kept in remission with a combinatory approach (combined antiretroviral therapy, *“cART”*) [12] is the infection with the human immunodeficiency virus (HIV). As HIV itself undergoes a microevolution due to the high mutagenesis by virus reverse transcriptase [13] it took decades to find an adequate multi-target treatment. And even with cART, the development of drug resistances especially for nucleotide reverse transcriptase inhibitors (NRTI) is still a major problem [14]. Due to the complexity of cancer, it can be anticipated that more sophisticated combinatory approaches are needed. An example for such a concept is CUSP9 where multiple drugs that are approved for non-cancer indications are combined as a treatment approach for recurrent glioblastoma [15–17]. The combination of compounds can lead to a broader effect on different tumor subtypes which may reduce chances of relapses or keep the tumor in a progression free state [18].

### Genome and metabolism stabilizing antitumor therapy (GMSAT)

**Fig. 1:**
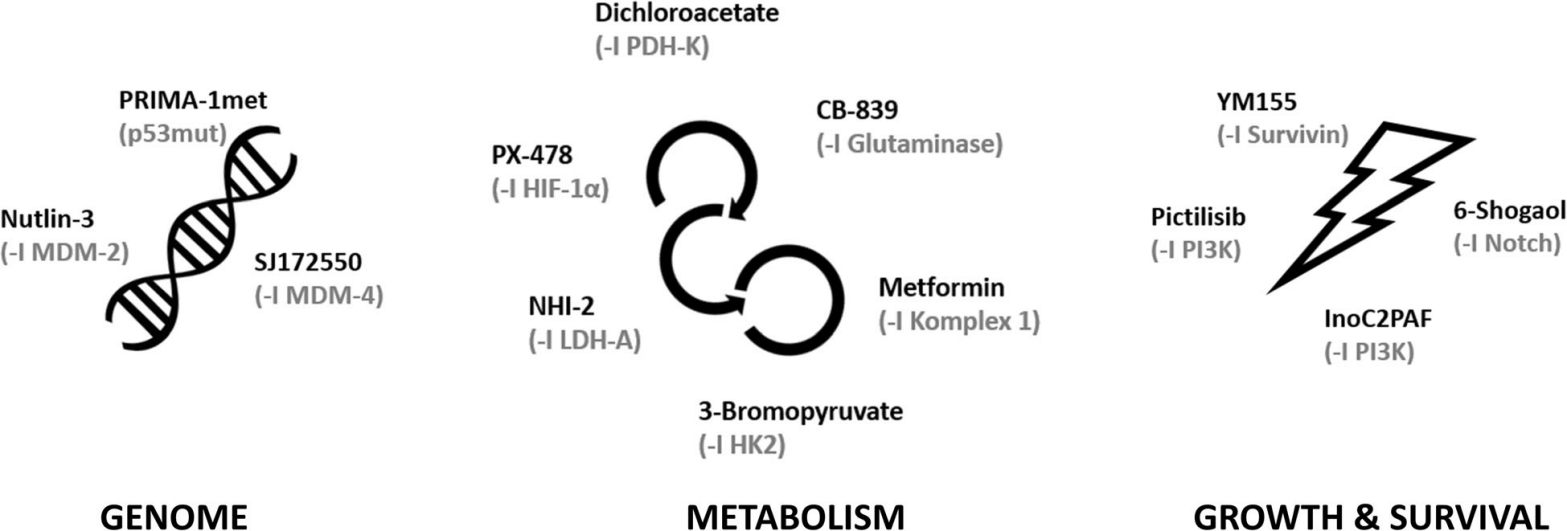
13 genome, metabolism and growth−/ survival targeting agents according to the GMSAT concept as well as Cisplatin as a reference to conventional chemotherapy are illustrated with their respective target structures in brackets. “**-I** “stands for inhibition

The here presented combinatory approach aims to counteract the tumor microevolution by targeting the genome, tumor metabolism as well as growth and survival (Fig. 1). PRIMA-1met and Nutlin-3 are two compounds targeting p53 which is often referred to as the “guardian of the genome” [19]. PRIMA-1met binds and reactivates mutated p53 [20] whereas Nutlin-3 increases p53 levels by disrupting the p53-MDM2 interaction and thereby inhibiting its degradation [21]. Likewise, SJ172550 counteracts the p53-MDM4 interaction which also leads to elevated p53 levels [22]. Compounds that modulate metabolism include Dichloroacetate (DCA) which aims to reverse the Warburg effect via activation of pyruvate dehydrogenase (PDH) by inhibition of pyruvate dehydrogenase kinase, promoting the entry of pyruvate into tricarboxylic acid cycle [23]. Other important metabolism targeting compounds used for our study are the hypoxia-inducible factor 1α (HIF-1α) inhibitor PX-478 (Koh et al. 2008), Metformin, which inhibits complex 1 of the respiratory chain [24], the inhibitor of lactate dehydrogenase A (LDH-A) NHI-2 (Allison et al. 2014) and the hexokinase 2 (HK2) inhibitor 3-Bromopyruvate (Ko, Pedersen, and Geschwind 2001). Another important energy source in cancer is Glutamine metabolism [25] which is targeted by the Glutaminase inhibitor CB-839 [26]. Finally, compounds targeting growth and survival are the survivin inhibitor YM155 [27], the phosphatidylinositol 3-kinase (PI3K) inhibitor pictilisib/GDC-0941 [28], InoC2PAF [29, 30] and the ginger derivate 6-Shogaol targeting the AKT/mTOR pathway [31].

### Screening for and evaluation of synergisms

In order to screen for potent synergisms, various successful methods have been tested and published recently [32, 33]. While some are relying on high throughput [34, 35] others are partially computerised to reduce the amount of actual experimental data points being investigated like the Feedback System Control [36–38]. There are also methods investigating synergism via mostly computerised analyses (Stochastic Searching Model, Statistical Model and Multi-Scale Agent-Based Model) [33, 39]. In literature, more than 10 different ways of defining synergism are described [40]. First referred to as the Loewe Additivity [41], quantification of synergistic drug interaction by the combination index (CI) is nowadays widely accepted. A precise method to estimate the specific dosages of fractional effects needed to calculate the CI, is the median effect method of Chou and Talalay that is derived from the mass action law [42, 43]. Quantification of synergisms via the CompuSyn software [44] based on multiple concentrations across the dose response curves is a well-established procedure [45].

## Methods

### Cell culture

MCF-7 breast cancer cells express p53 wild-type, are estrogen (ER) and progesterone receptor (PR) positive and express low levels of human epidermal growth factor receptor 2 (HER2) [46, 47]. MDA-MB-231 breast cancer cells that were originally isolated from a human breast cancer pleural effusion express a p53-mutation (R280K), are negative for ER and PR and express no amplification of HER2 [46, 48]. Both breast cancer cell lines were a kind gift of Göran Landberg (Sahlgrenska Cancer Center, University of Gothenburg, Gothenburg, Sweden) and were initially purchased from ATCC (Catalogue number: CRL-3435 and HTB-26). The primary colon cancer cell line HT-29 was isolated in 1964 by Fogh and Trempe. HT-29 cells carry a p53 mutation (R273H) and are deregulated for c-MYC [48]. HT-29 was a kind gift from Karsten Parczyk (Bayer AG) and initially purchased from ATCC (Catalogue number: HTB-38). All cell lines were routinely tested for mycoplasma contamination. For testing of mycoplasma contamination either PCR (GATC Biotech) or staining with Hoechst 33342 dye (Sigma-Aldrich, Steinheim, Germany) was conducted. HT-29 and MCF-7 cells were cultured in DMEM and the MDA-MB-231 in DMEM/F12 containing penicillin/streptomycin (100 U ml^− 1^), L-glutamine (DMEM: 584 mg l^− 1^, DMEM/F12: 365,1 mg l^− 1^) and 10% heat-inactivated fetal calf serum (FCS) at 37 °C in a humidified incubator with 5% CO2. Cells were harvested using 0.05% trypsin/0.02% EDTA in PBS.

### Compounds

Fourteen compounds were used: Prima-1met, Nutlin-3, SJ 172550, YM155 (Selleck Chemicals, Houston, TX, USA), 6-Shogaol (Hölzel Diagnostika Handels GmbH, Cologne, Germany), Pictilisib (Absource Diagnostics GmbH, Munich, Germany), Ino-C2-PAF (1-O-octadecyl-2-O-(2-(myo-inositolyl)-ethyl)-sn-glycero-3-(r/s)-phosphatidylcholine) [29], PX-478 (Hölzel Diagnostika Handels GmbH, Cologne, Germany), DCA, Metformin-hydrochloride (Sigma-Aldrich, Munich, Germany), CB-839 (Selleck Chemicals, Houston, TX, USA), 3-Bromopyruvate (Santa Cruz Biotechnology, Dallas, Texas, USA), NHI-2 (Bio-Techne GmbH, Wiesbaden-Nordenstadt, Germany) and Cisplatin (Cayman Chemical Ann Arbor, MI, USA). 3-Bromopyruvate, Cisplatin, Dichloroacetate, Metformin, PRIMA-1-met, PX-478, YM155 and Ino-C2-PAF were solved in distilled water. Dimethyl sulfoxide (DMSO) was used to solubilize 6-Shogaol, CB-839, NHI-2, Nutlin-3, Pictilisib and SJ-17255. Finally, DMSO concentration was kept under 0.6 μl per well (0.6%).

All data collected in this study can be found in the additional file (Additional file 1). This includes all data produced for dose response curves and all combination experiments.

### Cell viability assay and cell proliferation assay

0.5 × 10^4^ MCF-7, 1.5 10^4^ HT-29 and 1.5 10^4^ MDA-MB-231 cells per well were seeded in flat bottom 96-well plates. After 24 h and reaching a cell-confluence of approximately 50%, the respective compound or combination was added. As a negative control, cells were cultured in the presence of 0.6% DMSO. However, we could not detect any differences in cell viability between 0.6% DMSO and no DMSO. After 48 h of further incubation, either MTT assay (3-(4,5-dimethylthiazol-2-yl)-2,5-diphenyltetrazolium bromide, a tetrazole assay, Bio-Techne GmbH, Germany) or SRB (Sulforhodamin B) assay were applied. The MTT assay was performed according to the manufacturer’s instructions. For the SRB assay, cells were treated with 10% trichloroacetic acid (w/v) and stained with 0.06% SRB in 1% acetic acid for 30 min. Cells were then repeatedly washed using 1% acetic acid (v/v) followed by dissolution in 10 mM Tris (pH 10.5). Protein mass was monitored using a microplate reader at an optical density of 492 nm. All experiments were performed at least with two replicates in three independent experiments.

Dose response curves were obtained for 14 compounds using GraphPad Prism statistical analysis software 7.05. EC_50_ of the respective compounds was determined via nonlinear regression.

### Minimalistic drug interaction screening (MDIS)

MCF-7 and HT-29 cells were treated with 14 single and their 91 pairwise combinations at dosages of approximately EC_25_. All experiments were performed at least with three biological and two technical replicates. Thus, for one cell lines we produced about 909 data points (303 per biological replicate). The conjectured synergistical potency (CSP) of a combination was quantified by adding up the effect of the single compounds and subtracting the result from the combination’s effect. E.g.: Single dose A: 20% cell viability-reduction, single dose B: 10% cell viability-reduction and the combination of A and B exhibit cell viability-reduction of 37%. Thus, the combination of A and B reduces the cell viability 7% more than it is expected from simply adding up the effects of the single compounds (CSP = 7). Analyses were performed with Graph pad prism and Microsoft Excel.

### Confirmation of synergism

Synergism predicted by MDIS was evaluated with three to seven concentrations as suggested by Chou and Talalay [49].

MCF-7 and HT-29 cells were treated with the respective combination of compounds at a constant EC_50_:EC_50_ ratio as well as the same concentrations of each drug individually. Significant differences between single compound viabilities and combination viability was assessed by unpaired t-test. Only concentrations with p-values ≤0.05 for both compounds were considered as significant and marked by an asteriks (*) in the figures.

The combination indices (CI) were calculated using the CompuSyn software [44]. The CI is a quantitative value for the synergism of a drug combination at specific concentrations. A value below 0.3 indicates a *“strong”*, 0.3–0.7 a *“robust”* (originally referred to as “synergism” by Chou and Talalay), 0.7 to 0.85 a *“moderate” and 0.85 to 0.9 a “slight”* synergism. Values from 0.9 to 1.1 show an *“additive”* effect and a CI above 1.1 indicates *“antagonism”* [50, 51]. The CI was calculated as follows:

$$ CI=\frac{(D)_1}{(Dx)_1}+\frac{(D)_2}{(Dx)_2} $$

In the numerators, (D)_1_ and (D)_2_, are the concentrations of drug 1 and drug 2 in the drug-combination which have a certain effect on cell viability (x %). In the denominators, (Dx)_1_ and (Dx)_2_, stand for the concentration of each drug alone (drug 1 or drug 2) that is necessary to obtain the same effect (x %) as the drug-combination (drug 1 and drug 2). The concentrations (Dx)_1_ and (Dx)_2_ were calculated by CompySyn referring to individual cell-viability data of the concerning compounds. To enhance rigidity, (Dx)_1_ and (Dx)_2_ were predominantly generated via direct experimental data points. This way, potential calculation errors are ruled out as suggested by Zhao et al. [45]. To produce the median effect plots the following equation was used:

$$ {D}_x={D}_m{\left[\frac{fa}{1- fa}\right]}^{1/m} $$

Dm is the median effect dose, m counts for the slope of the median-effect plot and fa stands for fraction affected.

## Results

### Three-step concept to identify synergisms between selected compounds

**Fig. 2:**
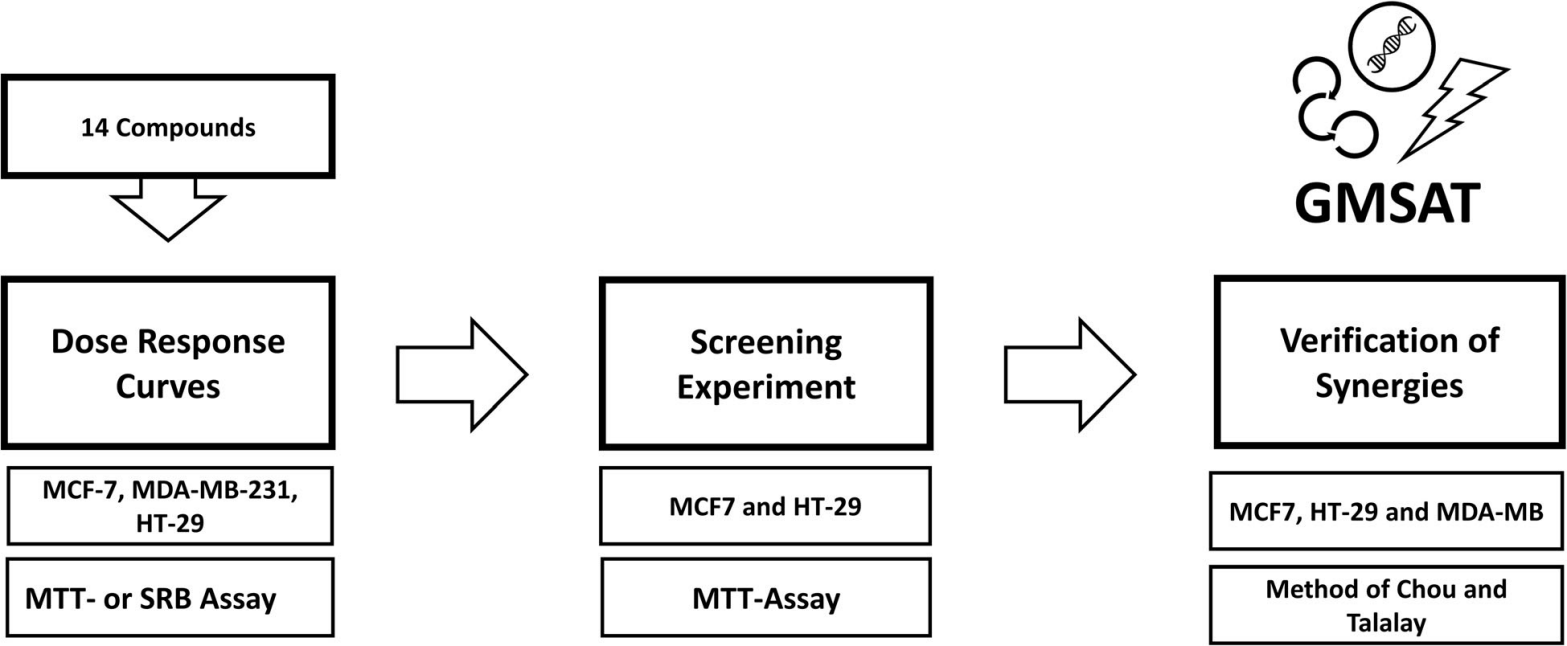
14 compounds were selected and analysed using MTT- or SRB Assay in HT-29, MCF-7 and MDA-MB-231 cells in order to obtain dose response curves and EC_50_. A minimalistic drug interaction screening (MDIS) was applied to detect synergies in the 91 possible combinations. The combinations with the most synergistic potential were then further verified

In this work, we applied the following three steps to identify synergisms between the compounds for our combinatory approach (Fig. 2).

1. Dose response curves aiming to detect the single drug effect in cancer cell lines and calculate fractional effects like EC_50_ or EC_25_.
2. The minimalistic drug interaction screening (MDIS) to identify potential synergies.
3. Verification by the method of Chou and Talalay to reliably prove the projected synergisms.

Following these steps, we identified 27 potential synergisms in MCF-7 (29.7%) and 19 in HT-29 (20.9%) of the 91 pairwise combinations. A selection of combinations was further analysed by the method of Chou and Talalay.

### Dose response curves in MCF-7, MDA-MB-231 and HT-29 cells

**Fig. 3:**
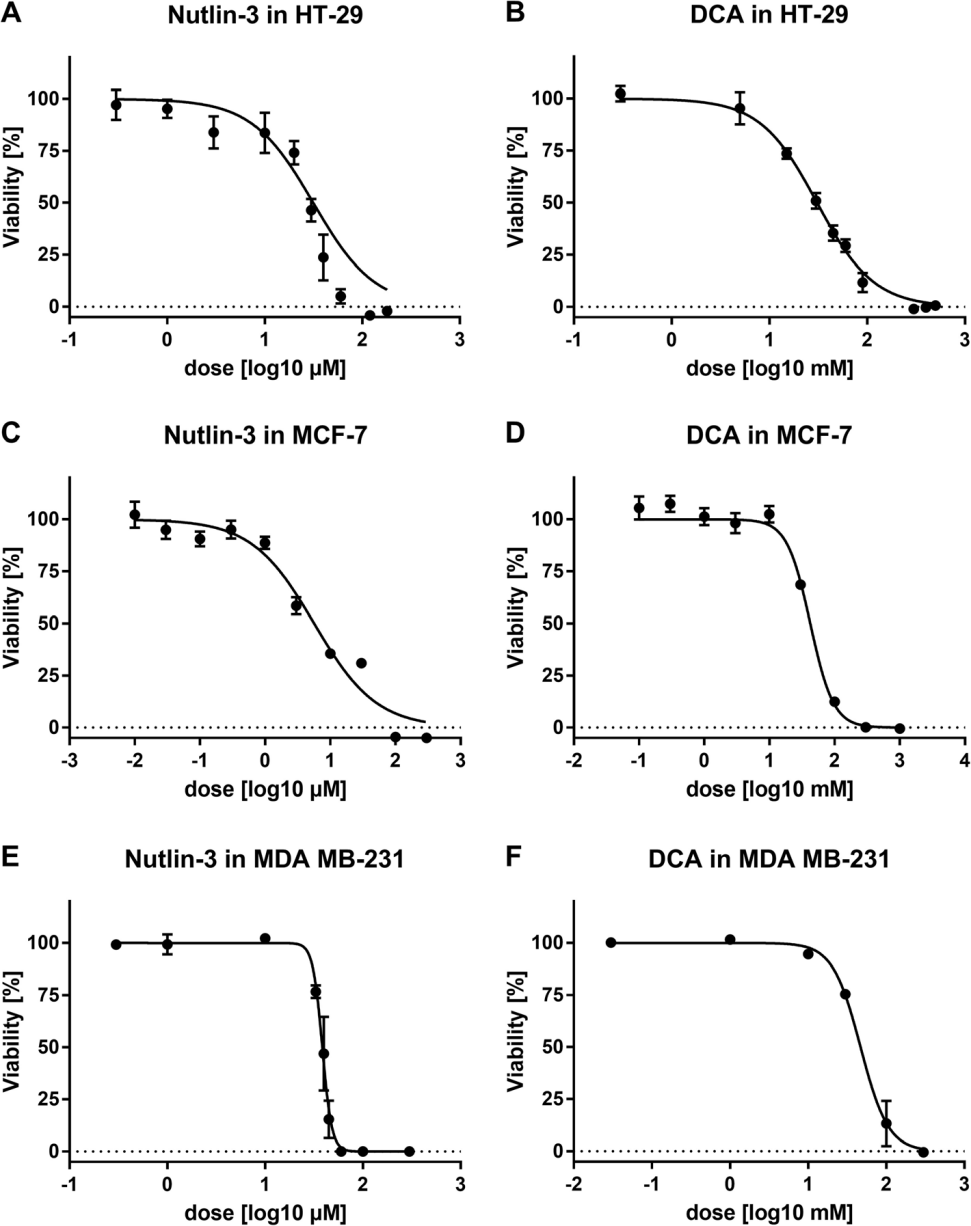
dose response curves Cells were seeded into a 96 well plate at a density of 1.5 (HT-29, MDA-MB) and 0.5 × 10^4^/well (MCF-7), incubated 24 h to a confluence of 50%, then cells were treated with increasing concentrations of the 14 selected drugs for 48 h. Viability was assessed using the MTT-Assay and curves were obtained using the four-parameter variable slope function of Graphpad Prism. Exemplarily the resulting curves for Nutlin-3 and DCA are shown for the three cell lines

Dose response experiments were conducted in order to identify the dose range for MDIS and evaluate the antitumor effects of the single compounds in different cell lines. Therefore, MCF-7, MDA-MB-231 and HT-29 cells were cultivated for 24 h before being treated with increasing concentrations of the 14 different compounds (Fig. 1). After an additional cultivation period of 48 h, cell viability or protein mass were quantified using the MTT or SRB assay. In Fig. 3, we exemplarily illustrated the dose response curves of Nutlin-3 and DCA for all three cell lines. Furthermore, we calculated the median effective concentration (EC_50_) for all compounds with the help of GraphPad Prism (Table 1). Data for all dose respond curves can be found in the Additional file 1.

**Table 1:** EC_50_ of the 14 compounds

Compound	Unit	HT-29 EC50	MCF-7 EC50	MDA-MB-231 EC50
**Prima-1met**	[μM]	64.9	18.1	71.1
**Nutlin-3**	[μM]	28.8	6.0	39.2
**SJ172550**	[μM]	15.4	15.6	229.5
**YM155**	[**n**M]	50.9	2.54	23.6
**6-Shogaol**	[μM]	29.7	148.9	503.5
**Pictilisib**	[μM]	19.8	0.16	532.8
**InoC2PAF**	[μM]	7.4	9.5	91.7
**PX-478**	[μM]	77.4	15.92	164.3
**DCA**	[**m**M]	34.9	40.7	48.7
**CB-839**	[μM]	3.3	6.0	13.3
**3-BP**	[μM]	15.6	110.7	554.8
**Metformin**	[**m**M]	6.9	13.4	100
**NHI-2**	[μM]	32.7	29.82	26.14
**Cisplatin**	[μM]	549.8	84.35	484.5

Cells were seeded into a 96 well plate at a density of 1.5 (HT-29, MDA-MB) and 0.5 × 10^4^/well (MCF-7), incubated 24 h to a confluence of 50%, then incubated with increasing concentrations of the 14 selected drugs for 48 h. Then, viability was assessed using the MTT-Assay and EC_50_ was calculated using Graphpad Prism

Overall, we observe that the triple negative breast cancer cell line MDA-MB-231 is the most resistant cell line requiring the highest dosages in 11 out of the 14 tested compounds. Although Prima-1met is intended to stabilize p53-mut, the strongest efficacy is shown in the p53 wild-type cell line MCF-7. YM155 is effective at very low concentrations at EC_50_ in a nM range in all three cell lines.

### Minimalistic drug interaction screening (MDIS)

**Fig. 4:**
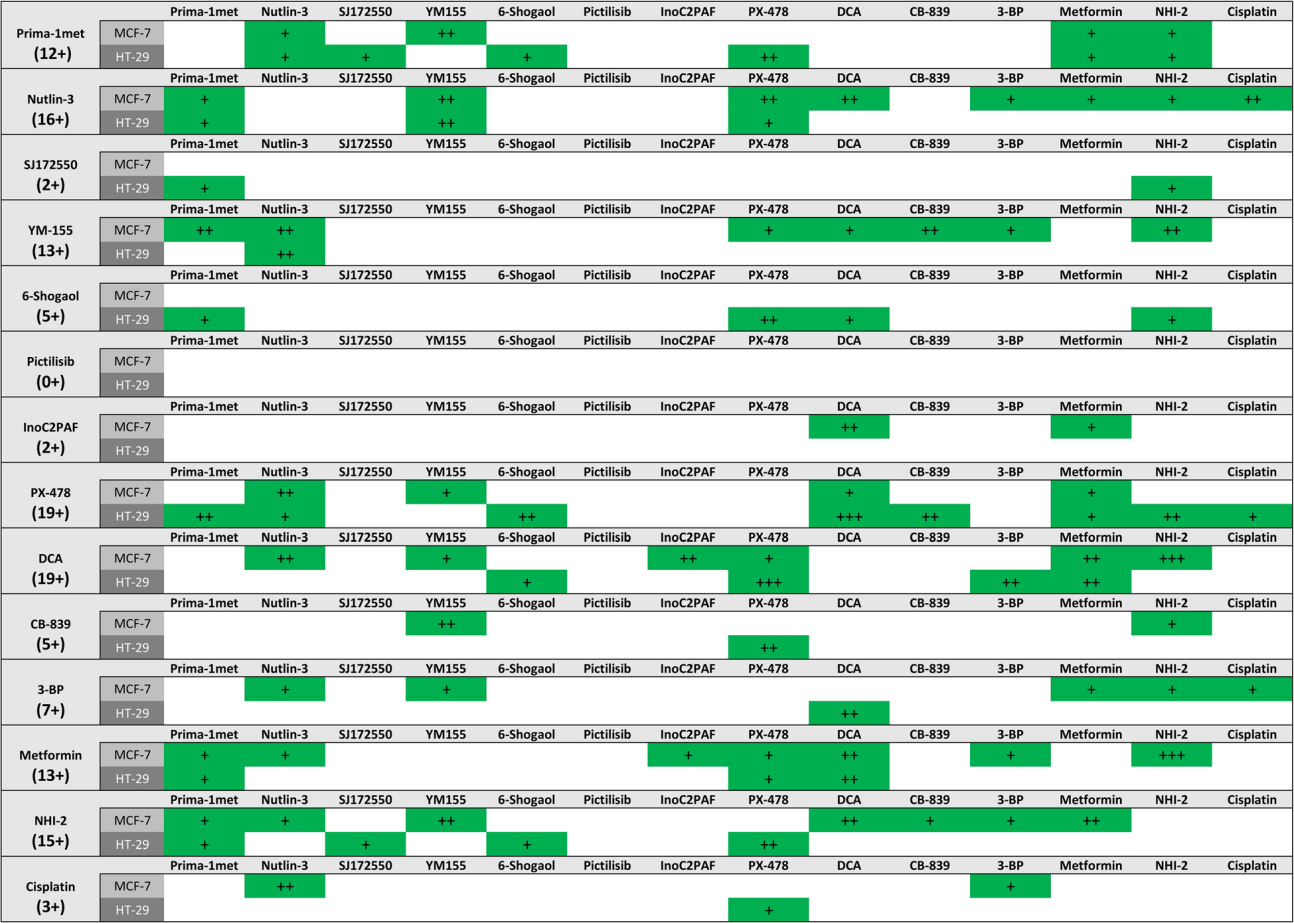
minimalistic drug interaction screening. HT-29 and MCF-7 cells were seeded into a 96 well plate at a density of 1.5 (HT-29) or 0.5 × 10^4^/well (MCF-7) and incubated 24 h to a confluence of 50%. Then, cells were incubated with 14 single compounds and the respective 91 combinations at a concentration about EC_25_ for 48 h. Viability was assessed using the MTT-Assay and the CSP (conjectured synergistical potency) values were calculated. CSP of a combination was quantified by adding up the effect of the single compounds and subtracting the result from the combination’s effect. All CSP values above ten are highlighted in green. Values between ten and 15 are marked by one plus (+), between 15 and 25 by two plus (++), greater than 25 by three plus (+++) and referred to as “possible”, “likely” and “very likely” synergism respectively. The number of total “+” is given in the first column below the name of the compounds and summarizes the number and strength of projected synergistic interactions

To identify synergistic actions of compound combinations, we developed a minimalistic drug interaction screening (MDIS). For this experiment, HT-29 and MCF-7 cells were treated with 14 different compounds in all 91 possible pairwise combinations. In this approach, dosages of approximately EC_25_ were used for all compounds. The conjectured synergistical potency (CSP) of a combination was quantified adding up the effect of the single compounds and subtracting the result from the combination’s effect (c.f. Material and Methods). We applied this rather simple mathematical approach not to prove synergisms, but to narrow down the number of effective combinations. The overall average standard deviations in MDIS were 7.5% for MCF-7 and 10.6% for HT-29 respectively. CSP values above 10 were chosen as a cut off for a ‘possible’ (+) synergism, 15 for a *“likely”* (++) and 25 for a *“very likely”* (+++) synergism (Fig. 4). Pure numerical values can be found in Additional file 1.

For HT-29 cells, a total amount of 19 synergistic projections out of the 91 combinations (20.9%) were predicted. Eleven of the latter were *“possible”* (12.1%), seven *“likely”* (7.7%) and one a “*very likely”* synergism (1.1%). For the p53 wild type breast cancer cell line MCF-7, a total of 27 combinations (29.7%) were identified, including 16 *“possible”* (17.6%), ten *“likely”* (11.0%) and one *“very likely”* (1.1%) synergism.

The highest CSP could be achieved in HT-29 for the combination of DCA + PX-478 which led to an average increase in inhibition of cell growth of 62.4% compared to the sum of the single dose effects determined for both drugs. Therefore, we performed deeper investigations with the combination of DCA + PX-478 in different cancer cell lines in a separate study. The second highest value was obtained for DCA + NHI-2 (43.4%) in MCF-7. Four combinations were projected to be synergistic in both cell lines: Nutlin-3 + YM155, DCA + Metformine, DCA + PX-478 and Nutlin-3 + PX-478.

### DCA, PX-478, Nutlin-3 and NHI-2 exhibit highest potential for synergistic interactions in MDIS

There were substantial differences in the count of potential synergies and their strength for the 14 compounds. The total number of “+” attributed to a compound by MDIS illustrates the synergistic potential of a compound since it summarizes quantity and quality of predicted synergistic interactions. With a total of 19 “+” the two compounds DCA and PX-478 have the highest synergistic potential. While PX-478 has the highest count of possible synergisms [12], DCA compensates a lower count [10] with stronger predictions (one vs. two *“very likely”* synergisms).

Additionally, with a total of 11 projections each, Nutlin-3 with 16 “+” and NHI-2 with 15 “+” show high synergistic potential. The lowest count of synergistic interaction was identified for the two PI3K-pathway targeting drugs Pictilisib and InoC2PAF with 0 and 2 predictions, respectively. YM155 had seven projections in MCF-7 and only one in HT-29. For 6-Shogaol, the opposite was the case: Five predictions in HT-29 and none in MCF-7.

### Analysis of the synergies by the method of Chou and Talalay

For further evaluation of these predicted synergisms according to the method of Chou and Talalay, we used the CompuSyn Software to calculate the combination indices (CI). The CI is a quantitative value for the synergism of a drug combination at specific concentrations. A value below 0.9 indicates synergism and the lower a CI, the stronger a synergism: A value below 0.3 indicates a “strong”, 0.3 to 0.7 a “robust” 0.7 to 0.85 a “moderate” and 0.85 to 0.9 a “slight “synergism. Values from 0.9 to 1 show a nearly “additive” effect and a CI above 1.1 indicates “antagonism”. Furthermore, significance in the differences between a combination and the respective single compounds was evaluated by unpaired T-test. We evaluated seven combinations projected by MDIS (Table 2). Five of the latter could be confirmed by the method of Chou and Talalay, while two combinations, PRIMA-1met + Nutlin-3 and Nutlin-3 +  3-Bromopyruvate did not reach significant p-values in detected synergisms (CI = 0.89 and 0.72 respectively). Since the combination of DCA + NHI-2 was promising in MCF-7 cells in both the screening trial (CSP = 43) and the method of Chou and Talalay (CI = 0.27), we further verified it in HT-29 (Table 2 and Fig. 6-C, D). Although it could not be detected by MDIS, we found the combination to be synergistic in HT-29 cells (CI = 0.50). Furthermore, we verified the most promising synergisms in MDA-MB-231 by calculating the CI-value using the dose response curves and equation of Loewe [41]. Thereby, we could confirm the top synergies DCA + NHI-2 (CI = 0.) and Nutlin-3 + PX-478 (CI = 0.62). Since we found a *“likely”* synergism between DCA + Nutlin-3 in p53 wild-type MCF-7 cells (Fig. 4), we checked the combination of p53mut binding PRIMA-1met + DCA in the p53-mutated MDA-MB-231 cells. Interestingly, a synergy exclusively found in MDA-MB-231 cells could be confirmed (CI = 0.78). After the evaluation of MDIS, we named synergies with CSP values between ten and 15 *“possible”,* 15 and 25 *“likely”* and greater than 25 *“very likely”* synergisms. Out of the seven verified synergies, we could prove all *“likely”* and *“very likely”* (4/4) but only two of the four possible synergisms. Thus, we detected eight (8.8%) and 11 (12.1%) *“likely”* and *“very likely”* synergisms in HT-29 and MCF-7 respectively.

**Table 2:** Verified synergies

Combination	Cell line	MDIS	C & T	MDA-MB
**DCA** **+ NHI-2**	MCF-7	+++	0.27*	0.62*
	HT-29	–	0.50*	
**Nutlin-3** **+ PX-478**	MCF-7	+++	0.33*	0.62*
	HT-29	+	0.63*	
**PRIMA-1met** **+ YM155**	MCF-7	++	0.34*	n.d.
**DCA** **+ Metformin**	MCF-7	++	0.51*	n.d.
**PRIMA-1met** **+ NHI-2**	HT-29	+	0.24*	n.d.
**Nutlin-3** **+ PRIMA-1met**	HT-29	+	0.89	n.d.
**Nutlin-3** **+ 3-Bromopyruvate**	MCF-7	+	0.72	n.d.

**Fig. 5:**
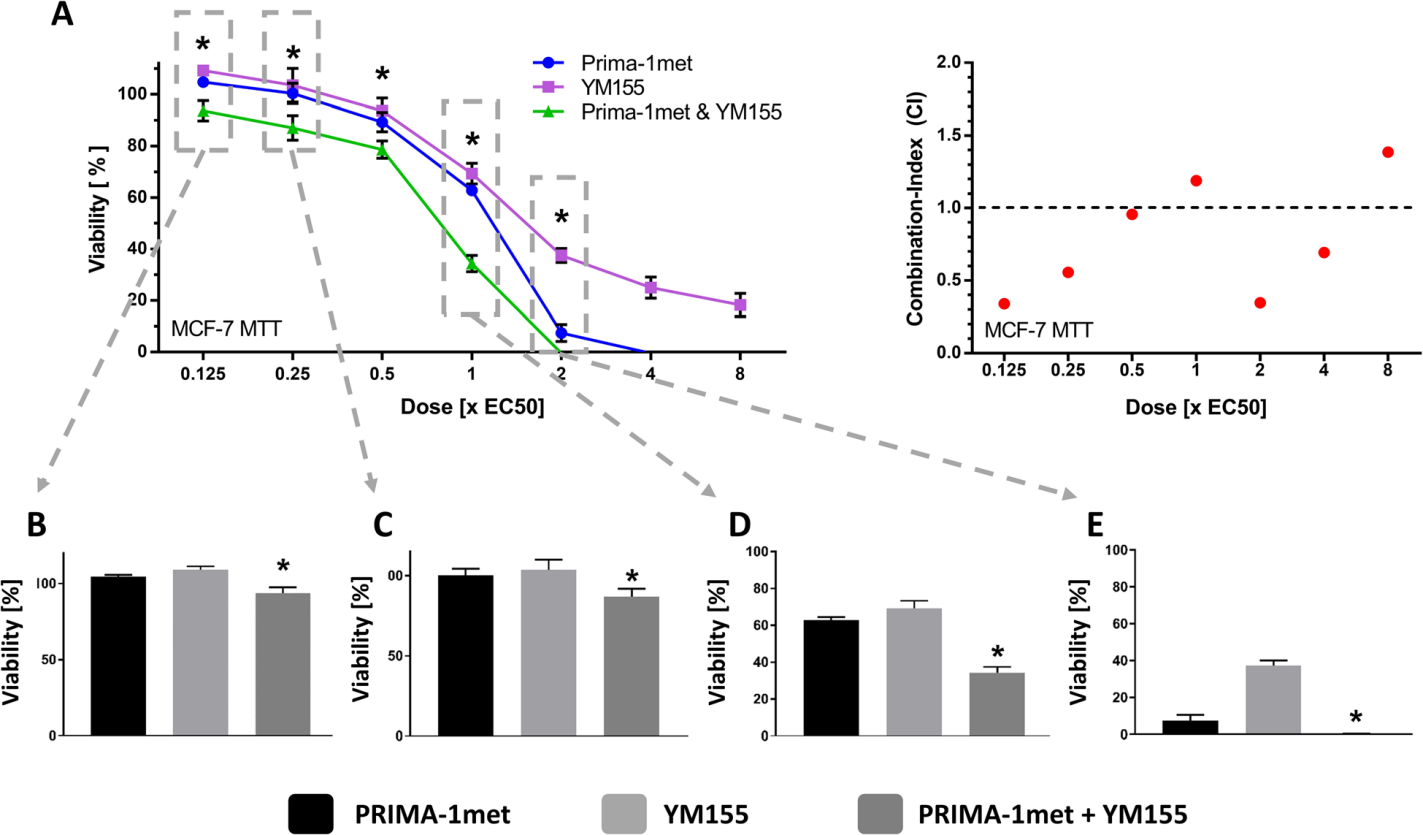
Synergy interpretation. HT-29 and MCF-7 cells were seeded into a 96 well plate at a density of 1.5 (HT-29) and 0.5 × 10^4^/well (MCF-7), incubated 24 h to a confluency of 50%, then medicated with increasing concentrations of PRIMA-1met, YM155 and their combination for 48 h. Cell viability was assessed using the MTT-Assay and curve was further analysed using Graphpad Prism. CI-Values were calculated by CompuSyn and illustrated with red dots in the diagram on the right. Each dot corresponds to the respective combination shown in the graph to the left. CI-values underneath the dashed line (< 1) imply a synergism. Viability data were also illustrated in a bar-chart design at 0.125x (B) 0.25x (C), 1x (D) and 2x EC_50_ (E)

**Fig. 6:**
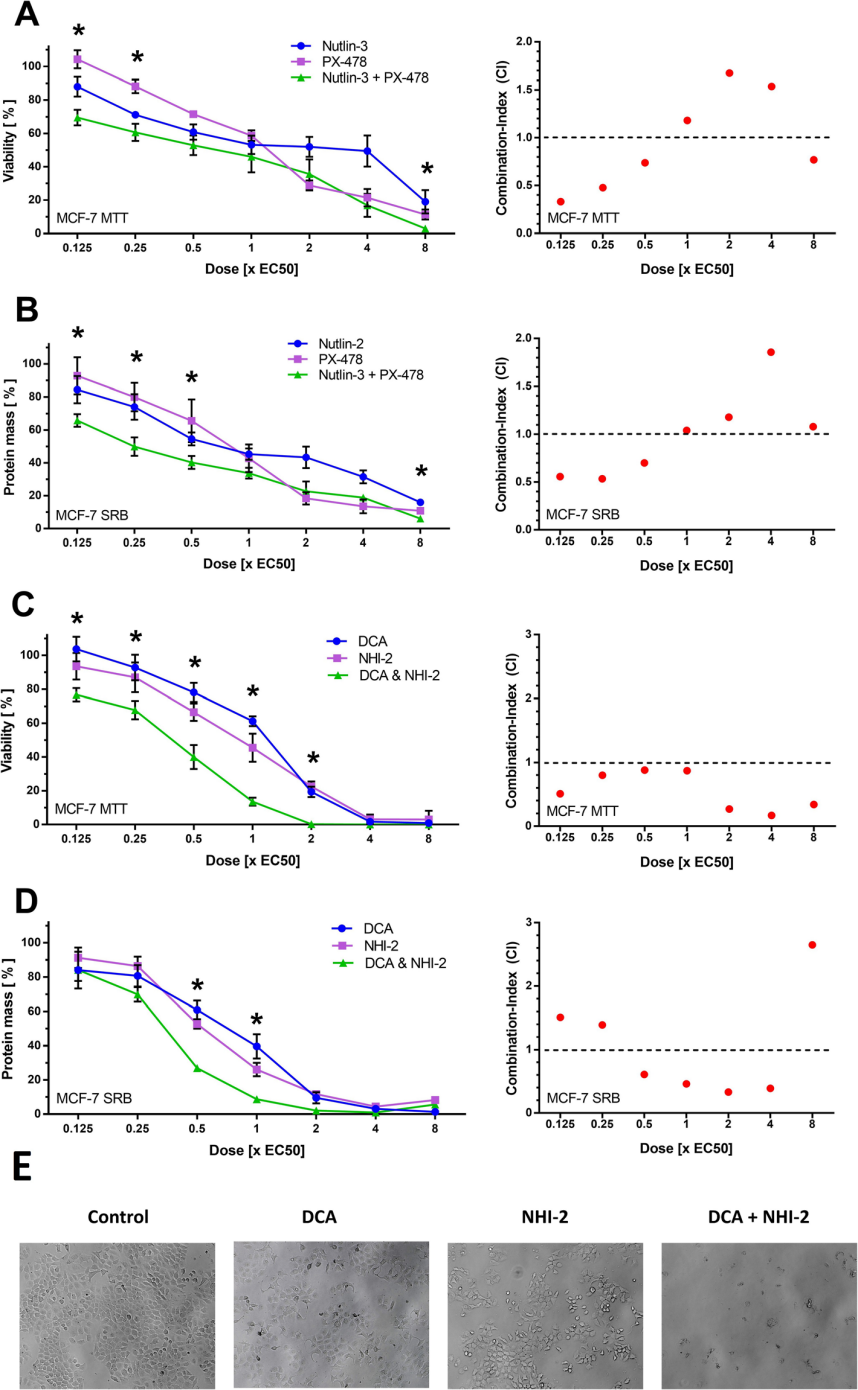
Nutlin-3 + PX-478 and DCA + NHI-2. MCF-7 cells were seeded into a 96 well plate at a density of 0.5 × 10^4^/well (MCF-7), incubated 24 h to a confluency of 50%, then incubated with increasing concentrations of Nutlin-3, PX-478 and their combination (**a**, **b**) as well as DCA, NHI-2 and their combination (**c**, **d**) and) for 48 h. Then, viability was assessed using MTT assay (**a**, **c**) and protein mass was assessed using SRB assay (**b**, **d**). CI-Values were calculated by CompuSyn and illustrated with red dots in the diagram on the right. Each dot corresponds to the respective combination shown in the graph to the left. The effects of *EC*_*50*_*of* DCA, NHI-2 and DCA + NHI-2 on the cell confluency is illustrated on the bottom (**e**)

Table 2 shows the seven combinations that were selected for further verification by the method of Chou and Talalay. The third column shows the predictions by MDIS: **+** indicates a “possible” **++** a “likely”‚ **+++** a “very likely” synergism and **–** no synergism. The respective best CI-values calculated by the method of Chou and Talalay (C & T) are listed in the fourth column. They were marked with an * if unpaired T-test was significant for the respective concentration of the combination in comparison with the single compounds. A CI-value indicates the quality of a synergism at a specific concentration. A value below 0.3 indicates a *“strong”*, 0.3 to 0.7 a *“robust”* and 0.7 to 0.85 a *“moderate”* synergism. In the case of MDA-MB cells, CI-values were calculated by the method of Loewe with the help of the earlier obtained dose response curves and Graphpad Prism. The resulting CI-values are listed in the fifth column. Combinations that were not analysed in MDA-MB-231 cells are marked with n.d. Out of the seven verified synergies, we could prove all “likely” and “very likely” (4/4) but only two of the four “possible” synergisms (two combinations had no significant CI-value below 0.9). Details concerning the combinations (the complete dose response curve and all the respective CI-values) can be found in Figs. 5 and 6 as well as in the Additional file 1.

### Interpretation of the combination index

When analysing drug interactions, looking at certain concentrations alone may lead to a false interpretation of synergism [42, 45]. The example of the synergism between PRIMA-1met + YM155 illustrates the principle of the CI-value interpretation (Fig. 5). At first sight, the combination of Prima-1met + YM155 shown in Fig. 5-D seems to exhibit stronger synergistic effects compared to lower dosages presented in Fig. 5-B. Contrarily to that assumption, the opposite is the case: 5-B shows indeed a *“robust”* synergism (CI = 0.34) while the effects shown in Fig. 5-D are not even *“additive”* (CI = 1.19). The explanation for this counter-intuitive finding is that doubling the single doses of PRIMA-1met + YM155 in EC_50_ results in a much stronger effect than the combination of both drugs at EC_50_ (Fig. 5-D, E). Therefore, one can conclude that the shape of and position on the curve is important to accurately describe and interpret synergisms. The easiest method to interpret synergistic effects of these curves consists in doubling the fractions of EC_50_. As a result, the CI calculations are mainly based on experimental data and can easily be interpreted by studying the curve progression. This method also helps minimizing errors that might occur with mathematical dose fitting [45].

### The combinations of Nutlin-3 + PX-478 and DCA + NHI-2 act synergistically in MCF-7, MDA-MB and HT-29 cells

The combination Nutlin-3 (inhibition of MDM-2) + PX-478 (inhibition of HIF-1α) was predicted to be synergistic by MDIS for HT-29 and MCF-7 cells. Via the method of Chou and Talalay, we analysed this synergism over the whole dose response curve. Exemplarily, we show in Fig. 6a and b the dose response curves for the combination Nutlin-3 + PX-478 and the single compounds. Best CI-values were 0.33 for MCF-7 (Fig. 6a) as well as 0.63 and 0.62 for HT-29 and MDA-MB-231, respectively (Table 2). In the reduction of protein mass (Fig. 6b) as well as the reduction of viability (Fig. 6a) it was mainly synergistic at 0.125x, 0.25x and 0,5x EC_50_.

Further, we confirmed the synergism of DCA + NHI-2 (PDH activation and LDH-A inhibition) in all three cell lines (Fig. 6c and d for MCF-7 and Table 2 for HT-29 and MDA-MB-231). A *“strong”* synergism was identified for the cell line MCF-7 (CI = 0.27) whereas a *“robust”* synergism could be found in HT-29 (CI = 0.50) and MDA-MB-231 (CI = 0.62).

## Discussion

We present here a three-step concept to systematically screen for and reliably describe synergies between a high number of compounds at a minimal cost and time budget. With that concept, we identified five synergistic combinations of genome and metabolism stabilizing compounds of which Nutlin-3 + PX-478 as well as DCA + NHI-2 were found in all three cell lines MCF-7, MDA-MB-231 (breast cancer) and HT-29 (colon cancer).

In contrast to the here presented approach, Borisy and colleagues designed a sophisticated high-throughput robot-assisted approach where 30 antifungal drugs and their 435 pairwise combinations were screened for potential synergistic interactions. For their screening experiment, six different concentrations with two technical replicates were used, resulting in a total of 31,320 data points [34]. For 14 compounds the same experimental design would result in 6552 compared to 303 data points with MDIS. While this approach provides a substantial amount of valuable information, it is material, cost and time intensive. Thus, optimization in material use and number of conducted experiments is needed to make drug interaction research feasible for a broader range of work groups.

### Dose-ratio based screening

Yin and colleagues reviewed, how computational based approaches such as the Feedback System Control [37] or Stochastic Searching Model with an heuristic idea can help to minimize costs of mainly experimental approaches [32]. Both approaches incorporate different dose-ratios already in the screening process. This design respects the fact that compounds interacting synergistically at a specific dose ratio may be antagonistic at other ratios [35]. Consequently, a screening without different dose-ratios may fail to detect synergisms that have antagonistic, additive or just slightly synergistic effects in the tested dose-ratio. In the here presented minimalistic drug interaction screening, this phenomenon is reflected in the fact that DCA + NHI-2 has not been projected to be synergistic by MDIS in HT-29 but could be proved by the method of Chou and Talalay (CI = 0.50). The opposite accounts for PRIMA-1met + YM155 which is synergistic in low doses (e.g. 0.125x EC_50_) and antagonistic at 8x EC_50_. Nevertheless, MDIS represents a substantial decrease in experimental scope: If for example three concentrations (e.g. EC_25_, EC_50_ and EC_75_) and all possible dose-ratios are used instead of one, the number of combinations increases from one to nine. Additionally, MDIS resulted in a total of 19 potential synergisms in HT-29 and 27 in MCF-7, a number that requires immense efforts to further verify and describe. Even when selecting only *“likely”* (++) and *“very likely”* (+++) synergisms, nine (HT-29) and 11 (MCF-7) combinations remain (Fig. 4). The focus on mechanistically interesting and most solid combinations in different cell lines is necessary to select most promising candidates. A dose-ratio based screening method is likely to detect even weak synergisms at an optimized dose-ratio and in that way it multiplies the number of projections. Therefore, we recommend the here presented cost-efficient design for projects that aim to evaluate interesting compounds of newly anticipated antitumor concepts for their synergistic potency. We recommend verifying the synergy over the entire dose-response curve at a constant dose-ratio before the determination of the optimal dose-ratios. Dose-ratio based screening might rather be appropriate for detailed analyses in order to optimize therapies of already implemented compounds [34].

### Synergy interpretation

After performing the three phases of the here proposed concept, we consider *“likely”* and *“very likely”* synergisms predicted by MDIS as the most relevant and solid results. In HT-29, we detected eight (8.8%) and in MCF-7 11 (12.1%) *“likely”* and *“very likely”* synergisms. Out of this group, we could confirm four of four tested synergisms (Table 2). In the case of *“possible”* synergisms, only two of four tested combinations could be confirmed. Nutlin-3 + PRIMA-1met and Nutlin-3 + 3-Bromopyruvate did reach synergistic CI values at some concentrations (CI: 0.89 and 0.72 respectively), but without significance. Furthermore, the CI-values over the whole dose-respond curve of these combinations were mainly additive or even antagonistic. Another “possible” synergisms detected by MDIS in MCF-7 is Metformin + Nutlin-3 which has already been described for mesothelioma cells by Shimazu et al. [52]. In general, *“possible”* synergisms might be worth examining as the *“robust”* synergistic effect between Nutlin-3 + PX-478 in HT-29 (CI = 0.63) illustrates (Table 2).

Out of the five detected and proven synergies, two top combinations were synergistic in all three cell lines. Nutlin-3 inhibits p53 degradation [21] while PX-478 modulates metabolism by inhibiting HIF-1α and thereby aerobic glycolysis [53]. While a mechanistic overlap is described in literature, we were – to the best of our knowledge - the first to detect this synergism. Lee and colleagues reported in 2009 that Nutlin-3 inhibits HIF-1α in a p53 dependent and vascular endothelial growth factor (VEGF) in a p53 independent manner [54]. These findings are supported by the fact that the Nutlin-3 + PX-478 showed the strongest synergism in the p53 wild-type cell line MCF-7 (CI = 0.33) compared to the p53 mutated cell lines HT-29 (CI = 0.63) and MDA-MB (CI = 0.62).

The second combination present in all three cell lines is DCA (PDH activation [55]) + NHI-2 (LDH-A inhibition [56]) which showed a *“strong”* synergism for the cancer cell line MCF-7 (CI = 0.27) and *“robust”* synergisms for HT-29 (CI = 0.50) and MDA-MB-231 (CI = 0.62). This combination has not been described in literature yet and is particularly interesting as both compounds target the “Warburg” effect [55], inhibiting the conversion of pyruvate to lactate and promoting its entrance into the tricarboxylic acid cycle. Out of the other four synergisms we were able to identify and prove, DCA + Metformine was already described thoroughly in literature [57].

### Validation of conjectured synergies

For the verification of the synergisms projected by MDIS, the widely accepted median-effect principle of the mass action law implemented in the method of Chou and Talalay was used [58]. To keep the transformation error low, we decided not to simplify our experiments by the overextended use of calculation and curve fitting for the determination of synergism [45]. In detail, we combined our compounds in a constant ratio of EC_50_ to EC_50_, stepwise doubling the dosages. We favour this method as the data necessary to calculate the CI-values have a solid empirical base. When a combination commends itself for further investigation, we suggest the following analyses:

1. The dose-ratio is crucial in the description of synergisms but cost and time expensive. Therefore, we suggest evaluating the most effective dose-ratios after a synergy has successfully been identified and proven.
2. To further evaluate the effectiveness of the detected combination, we recommend utilizing cell lines with different properties (e.g. p53 status) and or in different tumor entities [35].

### Limitations

In this work, we focused intensively on synergistic drug interaction in the detection of potential combinatory approaches. Synergistic effects are desirable, but additive effects or in some cases even compounds with slight antagonisms might be useful as well [18, 59]. For example, if the necessary single dose cannot be reached in vivo for pharmacodynamics reasons or dose limiting toxicity, a combination with a higher cumulative dose might result in a better outcome.

With respect to the genome and metabolism stabilizing antitumor approach, we conducted a systematic literature research to identify matching compounds. In contrast, large-scale prediction of drug combinations via different databases [18, 39] is another promising way of narrowing down the field of potential compounds. Generally, we based the calculation of the CI-values on substantial experimental data. If only half of the curve is measured experimentally while the other parts are calculated via curve fitting, changes in slope might be missed which could lead to false low CI-values [45]. Therefore, the amount of experimental data points and EC-range covered must be considered in the interpretation of the resulting CI-values.

### Clinical implications

To further evaluate promising combinations, taking already conducted clinical trials of the respective single compounds into account is important to identify potential obstacles and problems in the translational phase. When looking at DCA, “clinicaltrials.gov” does list 37 studies in the context of cancer and 81 studies in total. In one trial where patients with previously treated metastatic breast or non-small cell lung cancer were treated with DCA, the authors concluded that DCA should be used for patients with longer life expectancy and potentially in combination [60] (ClinicalTrials.gov Identifier: NCT01029925). PX-478 seems to be abandoned since the last clinical trial was conducted in 2010 (ClinicalTrials.gov Identifier: NCT00522652). In this phase 1 clinical trial PX-478 has been well tolerated in low doses with consistent HIF-1α inhibition in patients with advanced solid tumors [61]. A sufficient effect with well tolerated doses to commence with a phase 2 clinical trial seemed to be missing although a HIF-1α inhibition was achieved. As a conclusion, it can be stated that these two drugs are tolerated in the respectively needed dose while a convincing effect on cancer was missing. We believe that synergism is an important way to successfully include promising compounds like DCA and or PX-478 in the therapy of cancer. The synergisms with NHI-2 or Nutlin-3 identified in this study may be a solution in this context. For NHI-2 and Nutlin-3 no literature on clinical trials is available. However, it also seems that the effect of NHI-2 and Nutlin-3 on normal non-cancerous cells is tolerable. In vitro treatment with Nutlin-3 induced a significant cytotoxicity on primary CD19(+) B-CLL cells, but not on normal CD19(+) B lymphocytes, peripheral-blood mononuclear cells or bone marrow hematopoietic progenitors [62]. As for the molecular mechanism of NHI-2, Calvaresi et al. stated that LDH-A inhibition is unlikely to harm normal tissues [63].

## Conclusion

The here presented three-step concept proved to be cost and time efficient with respect to the resulting data at the example of our combinatory approach. *“Likely”* and *“very likely”* synergisms proved to be reliable predictions of MDIS after verification by the method of Chou and Talalay. The combination of Nutlin-3 + PX-478 as well as DCA + NHI-2 could be identified in all three cell lines. In vivo experiments are required to evaluate the potential of these combinations for clinical studies.

## Supplementary information

**Additional file 1.** Combination experiments, MCF-7 dose-respond-curves, HT-29 dose respond curves, MDA-MB dose respond curves and MDIS (minimalistic drug interaction screening). In this file all data concerning the combination experiments, the dose respond curves of the three cell lines and the MDIS can be found.

## Data Availability

All data generated or analysed during this study are included in this published article and its supplementary information file (Additional file 1).

## Abbreviations

CSP: Conjectured Synergistical Potency
CI: combination index
DCA: Dichloroacetat
GMSAT: Genome and Metabolism Stabilizing Antitumor Therapy
HIF-1α: hypoxia inducible factor α
MDIS: Minimal Drug Interaction Screening
PI3K: phosphatidylinositol 3-kinase
w/v: weight per volume
v/v: volume per volume
